# Right Hemisyndrome Secondary to Underlying Antiphospholipid Syndrome: A Case Report

**DOI:** 10.7759/cureus.40052

**Published:** 2023-06-06

**Authors:** Murtajiz M Raza, Marly S Oosterhof, Jordan Polo, Temiloluwa Njideaka-Kevin

**Affiliations:** 1 Department of Research, Avalon University School of Medicine, Willemstad, CUW; 2 Department of Family Medicine, Verpleeghuis Betèsda, Willemstad, CUW

**Keywords:** brain infarct, anticoagulant therapy, antiplatelet therapy, preeclampsia with hellp syndrome, anticardiolipin antibody, antiphospholipid syndrome, ischemic stroke, hemisyndrome, acute hemiplegia, antiphospholipid antibody syndrome (aps)

## Abstract

Antiphospholipid syndrome is an autoimmune disorder characterized by antiphospholipid antibodies, which can lead to both arterial and venous thrombosis. Neurological manifestations of antiphospholipid syndrome are diverse and can include stroke, seizures, and transient ischemic attacks. We present the case of an elderly patient with right hemisyndrome secondary to underlying antiphospholipid syndrome. This report aims to highlight the importance of recognizing antiphospholipid syndrome as a potential cause of neurologic deficits, precisely right hemisyndrome, and to emphasize the need for early diagnosis and appropriate management.

## Introduction

Antiphospholipid syndrome is an autoimmune disorder characterized by the presence of antiphospholipid antibodies, such as lupus anticoagulants, anticardiolipin antibodies, and anti-beta-2 glycoprotein 1 antibodies [1]. Antiphospholipid syndrome is known to cause both arterial and venous thrombosis, leading to a wide range of clinical manifestations. Neurological involvement in antiphospholipid syndrome varies from arterial etiology to venous and can occur in up to 30% of cases and may present as stroke, transient ischemic attack, seizures, cognitive impairment, or movement disorders [2]. Hemisyndrome is a rare but recognized manifestation of antiphospholipid syndrome and related diseases, where half of the body experiences purely motor, purely sensory, or both deficits due to different causes. This was discussed in an article that explored the role of antiphospholipid antibodies in causing cerebrovascular pathology leading to hemisyndrome manifestations [3]. Here, we present a case of a patient with right hemisyndrome secondary to underlying antiphospholipid syndrome.

## Case presentation

A 61-year-old female patient was admitted to the Department of Neurology for sudden-onset weakness of the right side of her body. The weakness involved the right upper and right lower limbs, with associated numbness and tingling sensations. The face was spared from any acute motor and sensory deficit, the micturition reflex was normal, and the patient was able to swallow without difficulty. The patient had an intracranial vertebral artery (ICVA) disease two years ago before admission. After the episode, she noticed cognitive disturbances and slow speech; however, she still lived independently at home. Her past medical history was significant for antiphospholipid syndrome, multiple brain infarcts, hypertension, miscarriages, preeclampsia with hemolysis, elevated liver enzymes, and low platelets (HELLP) syndrome. She was also noted to have delusions two years prior to admission, in which she believed she actively gave birth to a baby. She was on oral Exforge® (amlodipine/valsartan) to control her hypertension and oral antiplatelet and vasodilator therapy with Aggrenox® (acetylsalicylic acid/dipyridamole) to prevent further thrombotic events; however, she was non-compliant with medications. The patient denied any recent trauma or infection.

**Figure 1:**
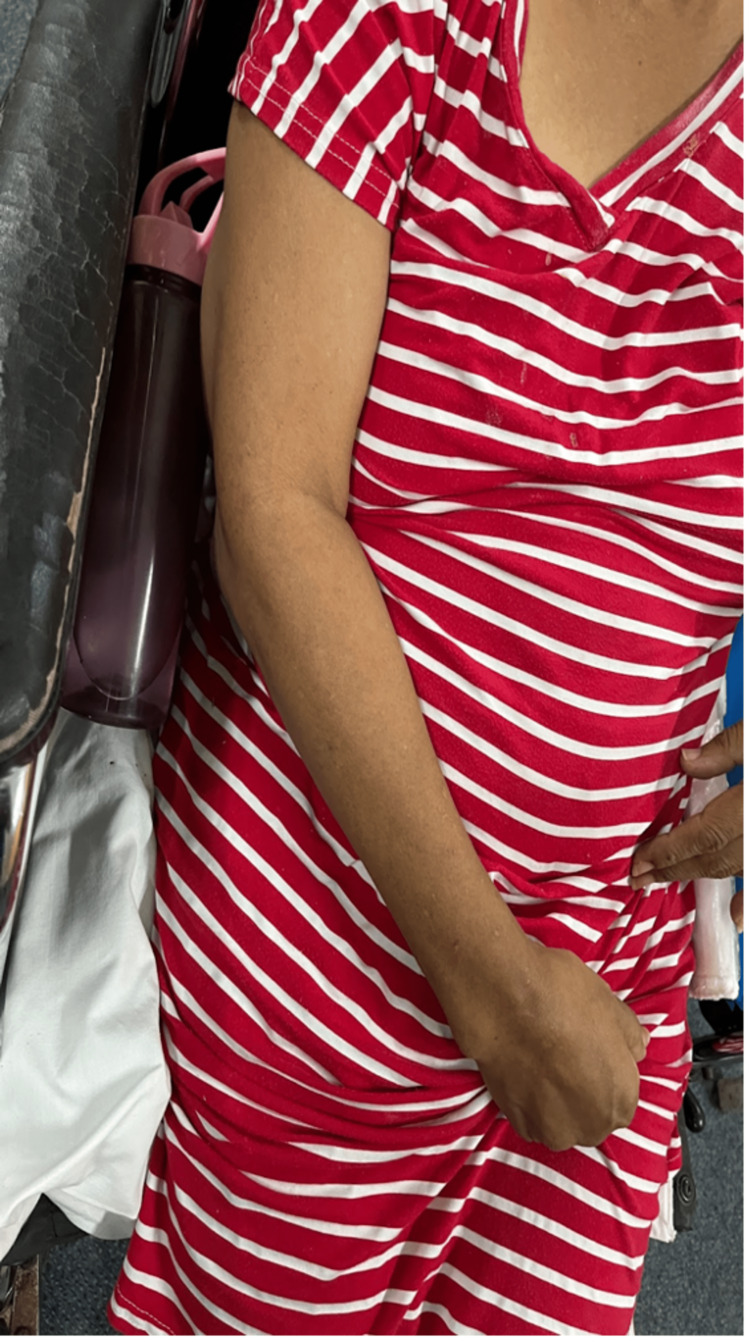
Atrophied right upper limb with spastic flexed right digits one through five

On examination, the patient had right hemiparesis with a Medical Research Council (MRC) grade of 0/5, no movement in right shoulder abduction, and 3/5 right shoulder adduction [4]. The forearm flexors were good but still a little weaker when compared to the left forearm, 3/5 when the patient moved her right arm up against resistance. Her right wrist was significant for no movement at all, 0/5. Her right digits (0/5) were spastic, limiting her range of motion (Figure 1).

**Figure 2:**
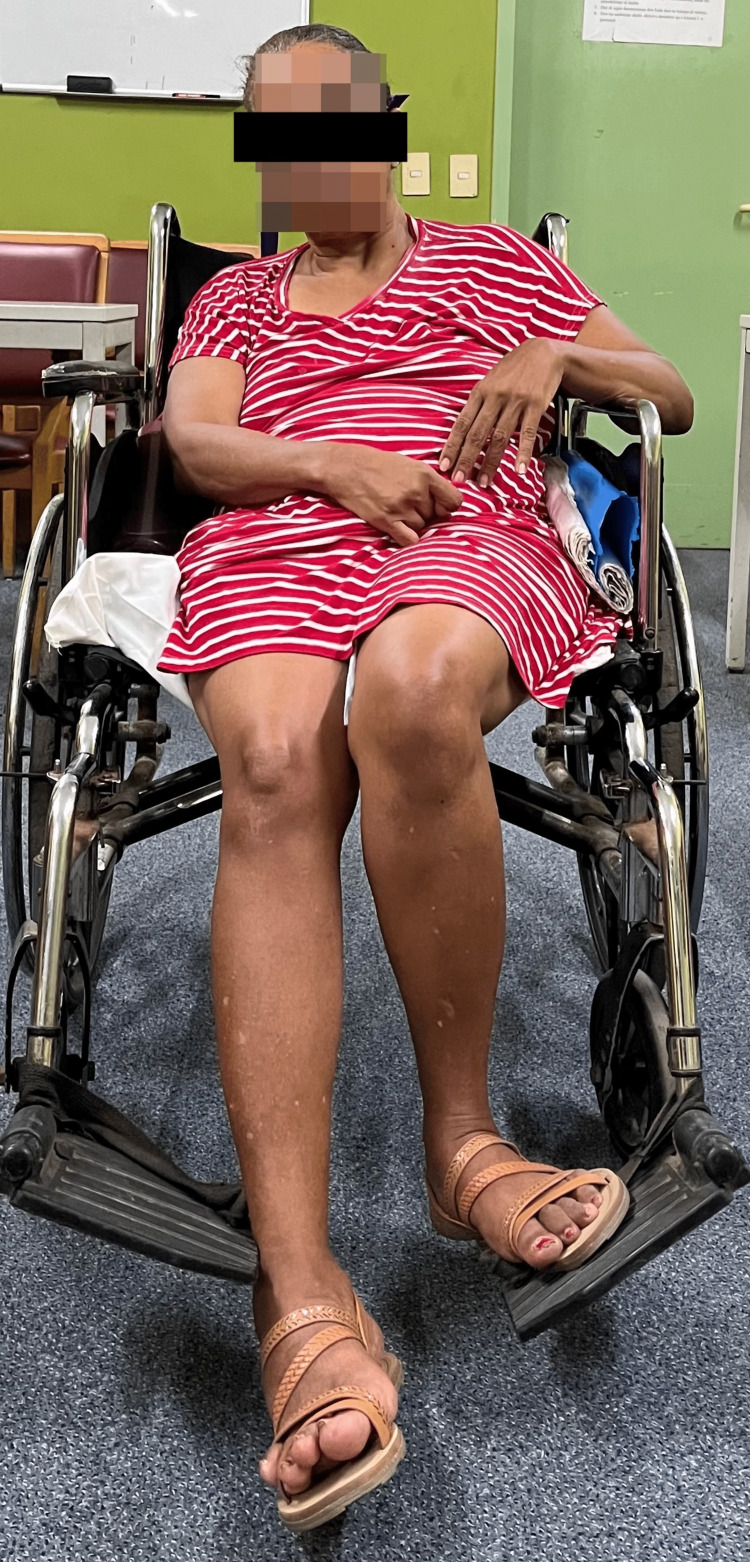
Right-sided weakness and right foot inversion at rest

The right knee was 4/5 for both abduction and adduction in the lower limb. The right ankle for both dorsi and plantar flexion was 2/5. The right foot was inverted on rest (Figure 2).

**Figure 3:**
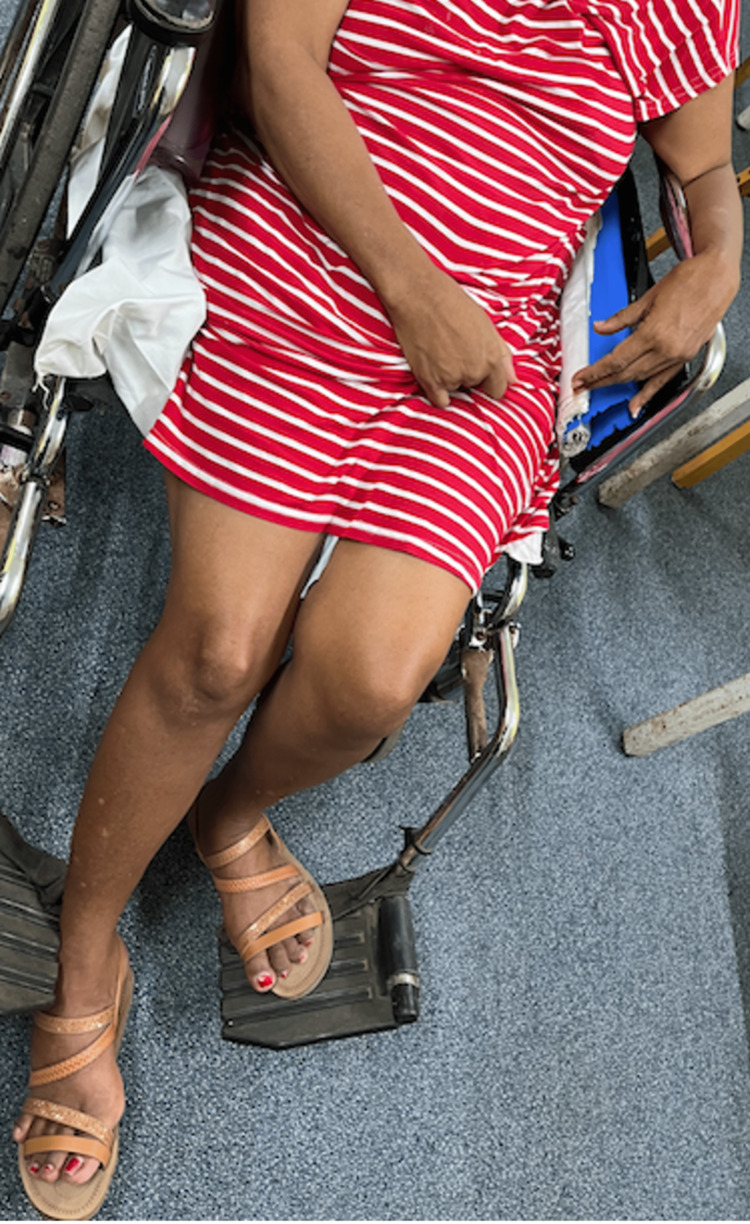
Atrophied right lower limb

The right biceps reflex was two plus, the right triceps reflex zero, the right brachioradialis reflex was two plus, the right patellar reflex zero, and the right ankle reflex two plus (Figure 3). Sensory examination revealed decreased sensation to pinprick and light touch on the right side of the body. All findings on the left upper and lower limbs were within normal range. The patient had no cranial nerve deficits, and the only significant vital sign was blood pressure (BP) of 180/100 mmHg.

Her most recent laboratory investigations showed higher glucose and C-reactive protein (CRP) levels than normal (Table 1). According to the patient's files, a brain computed tomography (CT) scan was done during her stay at the hospital and showed atrophy and severe leukoaraiosis, indicating a recent ischemia at the left internal capsule and left cerebral hemisphere. Unfortunately, we could not access any hardcopy or electronic imaging results as the patient was already discharged from the hospital and examined at a nursing home.

**Table 1:** Patient's lab values H: high; L: low; BUN: blood urea nitrogen; CPK: creatine phosphokinase; Na: sodium; K: potassium; CO2: carbon dioxide; CRP: C-reactive protein.

Blood Test	H/L	Patient’s Value	Normal Range	Units
Glucose	H	140	73-118	mg/dl
Urea		19	16-39	mg/dl
BUN		9	7-22	mg/dl
Creatinine		0.7	0.6-1.2	mg/dl
CPK		105	30-150	U/L
Na		136	136-146	mmol/L
K		4.0	3.5-5.1	mmol/L
Chloride		105	102-112	mmol/L
CO2		27	24-29	mmol/L
CRP	H	2.73	<0.80	mg/dl

## Discussion

The patient's clinical presentation and her history of antiphospholipid antibodies confirmed the diagnosis of right hemisyndrome secondary to underlying antiphospholipid syndrome. Right hemisyndrome refers to a combination of motor and sensory deficits involving the contralateral side of the body, typically caused by a lesion in the contralateral cerebral hemisphere [5]. In antiphospholipid syndrome, the presence of antiphospholipid antibodies can lead to thrombotic events in cerebral vessels, resulting in ischemic infarcts and subsequent neurological deficits [2].

Antiphospholipid syndrome can also lead to obstetric complications, as seen in our patient’s history. Antiphospholipid syndrome poses a significant risk in pregnancy as it can result in adverse outcomes such as recurrent miscarriages, fetal growth restriction, preeclampsia, preterm birth, and stillbirth. The antiphospholipid antibodies disrupt the normal function of the placenta and impair the maternal-fetal interface, leading to inadequate blood supply, thrombosis, and subsequent placental insufficiency. These pathological processes can compromise the fetus's growth and development and contribute to obstetric complications such as preeclampsia and HELLP syndrome, both of which were seen in our patient. Timely diagnosis, close monitoring, and appropriate management strategies, including anticoagulation therapy and regular prenatal care, are essential in mitigating the risks associated with antiphospholipid syndrome during pregnancy and improving outcomes for both the mother and the baby [6].

Antiphospholipid syndrome-associated right hemisyndrome treatment involves antiplatelet/anticoagulation therapy to prevent further thrombotic events, typically warfarin. Additionally, supportive management, including physical therapy and rehabilitation, plays a crucial role in restoring function and optimizing the patient's quality of life [7]. In our case, the patient was already on oral antiplatelet with Aggrenox®; and at the time of admission, the consulting team considered the patient not suitable for clinical evaluation but possibly suitable for polyclinical rehabilitation. She was mobilized with physiotherapy but had significant disbalance in the standing and sitting position.

## Conclusions

This case report highlights the importance of recognizing antiphospholipid syndrome as a potential cause of right hemisyndrome. Early diagnosis and appropriate management, including anticoagulation therapy and supportive care, are crucial in preventing further thrombotic events and optimizing patient outcomes. Healthcare professionals should maintain a high index of suspicion for antiphospholipid syndrome in patients presenting with neurological deficits, especially in the setting of a known autoimmune disorder and a history of thrombotic events.
